# Alternative Splicing of Fibroblast Growth Factor Receptor IgIII Loops in Cancer

**DOI:** 10.1155/2012/950508

**Published:** 2011-12-12

**Authors:** Klaus Holzmann, Thomas Grunt, Christine Heinzle, Sandra Sampl, Heinrich Steinhoff, Nicole Reichmann, Miriam Kleiter, Marlene Hauck, Brigitte Marian

**Affiliations:** ^1^Institute of Cancer Research, Department of Medicine I, Comprehensive Cancer Center, Medical University of Vienna, Borschkegasse 8a, 1090 Vienna, Austria; ^2^Division of Oncology, Department of Medicine I, Comprehensive Cancer Center, Medical University of Vienna, 1090 Vienna, Austria; ^3^Department for Companion Animals and Horses, Small Animal Internal Medicine, University of Veterinary Medicine Vienna, 1210 Vienna, Austria; ^4^Department of Clinical Sciences, College of Veterinary Medicine, North Carolina State University, Raleigh, NC 27607, USA

## Abstract

Alternative splicing of the IgIII loop of fibroblast growth factor receptors (FGFRs) 1–3 produces b- and c-variants of the receptors with distinctly different biological impact based on their distinct ligand-binding spectrum. Tissue-specific expression of these splice variants regulates interactions in embryonic development, tissue maintenance and repair, and cancer. Alterations in FGFR2 splicing are involved in epithelial mesenchymal transition that produces invasive, metastatic features during tumor progression. Recent research has elucidated regulatory factors that determine the splice choice both on the level of exogenous signaling events and on the RNA-protein interaction level. Moreover, methodology has been developed that will enable the in depth analysis of splicing events during tumorigenesis and provide further insight on the role of FGFR 1–3 IIIb and IIIc in the pathophysiology of various malignancies. This paper aims to summarize expression patterns in various tumor types and outlines possibilities for further analysis and application.

## 1. Introduction

Fibroblast growth factors (FGFs) are a large family of 23 ligands that serve crucial functions in embryonic development as well as in the adult organisms (for review see [1]). They mediate their signals via a small subfamily of 4 tyrosine kinase receptors (FGFR 1–4). This is a small number of receptors for a large group of ligands, but the system gains significant complexity from the formation of heterodimers as well as a high frequency of alternative splicing events [2, 3] that can be grouped in 3 categories. These categories are (1) the deletion of autoinhibitory domains close to the N- or the C-terminus of the proteins which produces more active and frequently oncogenic receptor variants [4, 5], (2) the IIIb and IIIc variants which are produced by alternative exon usage in the ligand-binding domain [2], and (3) soluble variants that come from exclusion of the exon that codes for the transmembrane region [6, 7]. All of these molecules display distinct biological activities. The IIIb and IIIc variations have been described for FGFRs 1–3 and probably have the strongest impact as they alter the ligand-binding portion of the affected FGFR [8]. This review will therefore focus on the IIIb/IIIc splice variants, their biological function, and the regulation of their formation especially in cancer. It will also describe novel methods for *in vitro* and *in vivo* monitoring.

## 2. Structure and Function of FGFRs

### 2.1. Expression and Splice Variants

The FGFR-genes consist of up to 20 exons that together code for a highly conserved protein domain structure [9, 10]. Each of the single FGFR genes can produce different mRNAs during the splicing process including variable exons, thus increasing the expressed protein diversity. 

Deletion of the Ig loop I (*α*-loop) has been described for FGFR1. This event creates a high-affinity oncogenic FGFR-variant that activated distinct signaling cascades [4, 11, 12]. Similarly, skipping of the most C-terminal inhibitory domain of FGFR2 has been described to result in a receptor molecule exerting transforming activity in human mammary epithelial cells [5]. The most common variation both in developmental processes and in cancer is the alternative splicing of Ig-loop III, however.

For the FGFRs 1–3, the Ig-loop III is encoded by two of the three consecutive exons 7–9, producing the domains designated IIIa, IIIb, and IIIc. The N-terminal half of Ig-loop III consists of the IIIa-sequence while the C-terminal half is formed by alternative usage of either the IIIb or the IIIc sequence creating the IIIb and IIIc isoforms of each receptor (see Figure  1 in [2, 3]). FGFR4 lacks an alternative exon and therefore does not have IIIb/IIIc splice variants [13]. Because the Ig-loop III is at the core of the ligand-binding site of FGFRs, the rearrangement profoundly alters the ligand spectrum of the receptor (Table 1). Overall the ligand-binding pattern of the IIIb variants is much more restricted than that of the IIIc variants [8].

### 2.2. Physiological Function of FGFRs in Development and Tissue Maintenance

FGFRs serve an essential role in embryology from gastrulation [14] to organogenesis as shown by extensive studies of gene expression patterns and genetic analysis using knock-out mouse models (reviewed in [1, 2, 15]). Knock out of several FGFs in specific mouse models causes an embryonic lethal phenotype or death at birth due to severe deficits in organogenesis. This underlines the essential role of FGF-signaling in embryonic development that is also mirrored with FGFR deletion. Specifically, knock-outs of FGFRs 1 and 2 are embryonic lethal, while FGFR3 and FGFR4 knock out mice are viable [2]. With regard to the IIIb/IIIc splice variants it is interesting to observe that specific deletion of the exons 8 or 9 produced distinct phenotypes—demonstrating distinct roles for each variant FGFR. For FGFR1, deletion of exon 9 (IIIc deficiency) is embryonic lethal while deletion of exon 8 leading to loss of the IIIb variant has no obvious phenotype [16]. For FGFR2, loss of the IIIb variant is lethal and deletion of the IIIc causes skeletal malformations [17–20]. 

Deeper insight in the specific roles of the IIIb and IIIc receptor variants of the FGFRs 1–3 is provided by analysis of their distinct spatial expression patterns. Extensive studies of mouse embryonic development demonstrate characteristic localizations of FGFR isoforms during organogenesis. This is observed throughout the body [17, 21], but is best investigated for bone and dental development [22, 23]. In general the IIIb forms of FGFRs 1–3 are expressed in epithelia while the IIIc variants are preferentially found in the mesenchyme [21, 24]. In concert with complementary expression patterns of FGF ligands, tissue-specific FGFRs mediate epithelial-mesenchymal tissue crosstalk during embryonic development. It was shown that FGFRs expressed in the epithelium or connective tissues are activated by FGFs secreted from the respective complementary tissue. 

The distinction is best observed for FGFR2 whose IIIb/IIIc choice is strictly tissue specific [24]. During developmental processes throughout the body, expression of FGF7-family members is observed in the connective tissue. The secreted FGF7 and/or FGF10 activate the epithelial-specific FGFR2-IIIb to induce a morphogenetic response in the epithelium [25–27]. This response includes induction of FGFs 8, 4, or 9, secretion of the factors from the epithelial cells and activation of IIIc-receptor variants in the mesenchyme [28] (Figure 2). There is some redundancy in the FGF family so that FGFs with similar receptor affinities can substitute for one another. However there are also examples of one specific FGF being essential at an organ site and severe phenotypes in case of a deficiency—for example, FGF10 in lung development (reviewed in [1, 2]). In adult tissues the specific expression of FGFR2 variants are maintained, enabling tissue interactions in wound healing and tissue repair. FGFs 7 and 10 are produced by dermal cells to stimulate reepithelialization through a paracrine mechanism [29–31]. 

 For FGFR3 the distinction is less clear as isoform expression is not strictly tissue specific. While the IIIb form is restricted to epithelia, the IIIc variant can be found in both the mesenchyme and the epithelium [33]. An example is the intestinal mucosa, whose main FGFR3 is FGFR3-IIIb [34]. In undifferentiated fetal colon, expression of FGFR3-IIIc has been observed, however [35]. FGFR3-IIIc expression would sensitize the mucosa cells to growth and survival signals from FGF18 [8]. This growth factor is a *β*-catenin target gene and expressed in the stem cell compartment at the bottom of colonic crypts [36, 37]. Similar observations come from the analysis of gene expression in the LT97 adenoma cell line we have established from human early colonic adenomas. When the total cell population was analyzed, the presence of FGFR3-IIIb, but not FGFR3-IIIc, could be demonstrated [38]. However, a subsequent study revealed the presence of a CD44-positive subpopulation with increased growth and survival capacity within the LT97 cell population [39]. This specific cell population overexpressed both FGFR3-IIIc and FGF18 as compared to the CD44-negative cells (unpublished observation).

## 3. Exon Switching in Malignant Progression

In the past several years evidence has accumulated that demonstrates a central role for deregulated FGF/FGFR signaling in a large number of malignant tumors (for review see [32, 40, 41]). Hyperactivation of FGFR-dependent signaling can be achieved by several mechanisms, including altered splicing of FGFRs 1–3 [32, 42]. Splice variation in FGFRs leads to expression of mesenchyme-specific FGFRs in epithelium-derived cancers and broadens the range of FGFs that can stimulate tumor cells. In most instances this enables autocrine stimulation of tumor cells by FGFs that are secreted by the epithelium to stimulate connective tissue cells in a normal physiologic context [8].

A FGFR2-IIIb to IIIc switch is related to increased invasiveness in bladder and prostate cancers [43]. In the prostate, the switch of FGFR2 replaces an IIIb receptor variant that exerts antitumorigenic activity [44, 45], with a protumorigenic IIIc receptor. It is therefore a marker of tumor progression and tumor invasiveness [42, 44, 45]. The FGFR2 IIIb to IIIc switch is also listed among the criteria of epithelial-mesenchymal transition (EMT) that confer a migratory, metastatic phenotype to several advanced carcinomas (reviewed in [46]).

For other receptors the contrast is less clear. However, FGFR1-IIIc has been upregulated in several carcinomas where it is regarded as a strong oncogene [47, 48]. In both nonsmall cell lung tumors and glioblastomas, the upregulated receptor permits autocrine stimulation by FGF5 [8]. The expression of FGFR1-IIIc also results in sensitivity to growth inhibition by a dominant negative FGFR1-IIIc construct—underlining the central role of this receptor [47, 49]. In prostate cancer as well as in TGF*β*-induced EMT FGFR1-IIIc is upregulated as an independent mesenchymal marker and the reciprocally downregulated IIIb receptor is FGFR2 [50, 51].

In colorectal cancers the reciprocally downregulated receptor is FGFR3-IIIb. Restoration of its expression inhibits growth under some conditions [52]. The mechanism involved is defective splicing that produces a nonsense transcript of FGFR3-IIIb, but not increased expression of FGFR3-IIIc. Frequently, FGFR3-IIIc expression is retained similar to the level in normal mucosa, however, which permits transduction of growth and survival signals mediated by FGF18, expression of which is upregulated in colorectal cancer [37, 38]. A dominant-negative FGFR3-IIIc construct, but not the respective FGFR3-IIIb mutant, inhibits growth and survival in colorectal tumor models *in vivo* and *in vitro* and also blocks colorectal tumor cell migration [38]. 

For several other tumor types investigation of FGFR IIIb/IIIc expression patterns are still ongoing. An interesting example was found in ovarian cancer cells most of which express both the IIIb and the IIIc forms of FGFR 2 and 3. By contrast immortalized ovarian surface epithelial cells express mainly the IIIc forms while FGFR2-IIIb is the main receptor type in nonimmortalized ovarian epithelial cells (unpublished observation). Even though not completely understood, this does indicate a high degree of plasticity in this cell type and seems to support the hypothesis that ovarian cancers may arise from mesenchymal fimbrial cells [53]. 

In contrast to carcinomas, very little is known of the role of FGF/FGFR signaling in the disease progression in soft-tissue sarcomas (STS). To date, there are fewer than 10 publications covering any aspect of FGFR signaling in STS. Research is hampered by the low incidence of these tumors in humans, so we have initiated comparative studies of STS in humans, dogs, and cats. The dog, with an STS incidence rate estimated to be 12-fold to 33-fold higher than the incidence in humans [54–56], is an excellent model of spontaneously arising sarcomas while feline injection site sarcomas may offer insight into the role of FGFR in the development of inflammation-related tumors [57].

Preliminary data have been collected by our research group from human and animal cell lines and patient tissues. We could identify all FGFRs and their splice variants in human and canine STS cell lines as well as in canine spontaneous tumor samples. Specifically, in human and canine STS cells FGFR1-IIIc is the FGFR with the highest transcript levels (Figure 3). 

 Feline sarcomas differentially express FGFRs depending upon the underlying cause of tumor formation—that is, injection site sarcomas versus spontaneous sarcomas (unpublished data). As these studies progress we will be able to address the question whether STS require alterations in FGFR expression patterns to achieve a metastatic phenotype and whether a “switch” between the FGFR IIIc or IIIb splice variants has an impact in this process.

## 4. Exogenous Regulation of the IIIb/IIIc Switch

Expression profiling in both cell lines and tissues indicates that the splicing choice for FGFR-IIIb and IIIc variants is strictly regulated. For FGFR2, the choice appears mutually exclusive and maintained even in the absence of a functional alternative exon [59]. This strict expression pattern is also supported by the observation that the splice switch resulting in more aggressive prostate and bladder tumors is also mutually exclusive [42]. Based on the strong tissue specificity and mutual exclusivity, this splice variant switch is regarded as one of the hallmarks of EMT [46]. As such, it is correlated with all the complex alterations producing EMT—activation of transcription factors such as snail, loss of E-cadherin, and upregulation of the WNT pathway [60, 61]. The splice decision may therefore be subject to regulation by any of the factors inducing EMT—transforming growth factor *β* (TGF*β*), WNT signaling, and activation of tyrosine kinase receptors, for example, by hepatocellular growth factor (HGF), or FGF family members [62, 63]. It has not yet been ascertained that the splice choice is a cause or consequence of EMT. 

Two pieces of information exist that link FGF signaling itself with alternative splicing. (1) In the prostate there are indications that loss of FGFR2-IIIb stimulation by its ligand FGF7 contributes to the switch—indicating that the activated receptor reinforces its own expression [64]; (2) in NBT-II rat bladder carcinoma cells and SVK14 human keratinocytes, exogenous FGF1 or FGF2 induced an FGFR2 IIIb/IIIc switch [65]. 

Isoform expression patterns indicate that the choice is made differently, or at least not as stringently, for FGFRs 1 and 3—with FGFR1-IIIc being upregulated in carcinomas without a concomitant loss of the IIIb variant [47, 48] and both FGFR3 variants being expressed in epithelia [33, 35]. This leaves open the question whether regulation of splicing is achieved by similar mechanisms for all 3 receptors.

In this context it is interesting that downregulation of FGFR3-IIIb expression in colorectal carcinoma cells is mediated by aberrant splicing that produces out-of-frame nonsense transcripts [66]. There is no concomitant upregulation of FGFR3-IIIc but an inverse relationship has been observed with FGFR1-IIIc and FGFR3-IIIb expression. siRNA-mediated FGFR1-IIIc knock down induced FGFR3-IIIb expression while FGFR3-IIIb overexpression suppressed FGFR1-IIIc [52].

## 5. Regulation of the IIIb/IIIc Switch by Endogenous Factors

At the transcript level, regulation of splicing is achieved by auxiliary cis-elements that bind regulatory proteins to either enhance or silence splicing of adjacent exons [67]. The cis-elements are thought to consist of short, conserved RNA sequences of typically 10 nucleotides in length that bind regulatory proteins with splicing enhancer and silencer properties. These elements can be located either in exons or introns and act alone or in clusters [68]. In addition, some silencers create secondary structures in the pre-mRNA that hinder recognition of neighboring splicing enhancers by regulatory proteins [69]. 

Most known splicing regulators are RNA-binding proteins (RBP), such as the serine/arginine-rich (SR) and heterogeneous nuclear ribonucleoprotein (hnRNP) family of proteins. These RBPs are fairly ubiquitously expressed, albeit with some differences in expression between tissues [70]. Detailed molecular characterization and structural modeling of the spliceosome and the analyses of RNA regulatory elements may help to clarify the complexity of alternative splicing regulation. 

For FGFR2 exon IIIb and exon IIIc splicing, previous studies identified a number of auxiliary cis-elements and RPBs that regulate the splice event ([71, 72] and references therein). In general the hnRNP H family of proteins has been shown essential for activation of exon inclusion, but in case of FGFR2 exon IIIc these proteins have been identified as silencers that repress exon inclusion [72]. This gene and context dependence of hnRNP function suggests that a complex series of RNA-protein and protein-protein interactions are involved in exon inclusion or exclusion. The details of these interactions remain to be discovered. 

A genome-wide high-throughput cDNA overexpression screen identified additional splicing factors that promote FGFR2 exon IIIb expression [73]. Among the splicing factors identified were two paralogous epithelial cell-type-specific RBPs termed epithelial splicing regulatory proteins 1 and 2 (ESRP1 and ESRP2) that are essential tissue-specific regulators of FGFR2 splicing. Furthermore, ESRP1/2 do not only regulate FGFR2 splicing, but a whole set of genes involved in EMT that induce the striking cellular changes—specifically loss of cell-cell adhesion and polarity and gain of migratory and even invasive properties. Overexpression and knock-down experiments indicate that their regulation is sufficient to induce EMT/MET-related splice patterns [74, 75]. As described above, EMT can be initiated, amplified, and modulated by overlapping signaling pathways like WNT and TGF-*β* via gene expression changes that led to the cells' phenotypic transitions [74, 75]. Recently ESRP1/2 have been shown to act as mastermind splice regulator, sproviding an additional layer of gene regulation that contributes to shape the EMT process [74, 75]. The answer as to how they are targeted by EMT-inducing pathway has not been reported to date.

Sequence and experimental analyses generated a predictive “RNA map” in which binding of ESRP1/2 either within or 5′ upstream of an alternative exon leads to exon skipping, whereas binding to the downstream intron leads to exon inclusion [74]. Applying such an “RNA map” on sequences of all FGFR IIIb and IIIc variants from human and other species, like dog and cat, may predict similar splicing regulation by ESRP1/2 as observed for FGFR2 exon IIIb (see Figure  3 in [74]). Such results must be validated by experiments with tumor cells of epithelial and mesenchymal origin that clearly demonstrate a connection between exon IIIb/IIIc usages of all FGFRs and expression of ESRP1/2 to address whether splicing is regulated by similar mechanisms for all 3 receptors. Indeed, our own observations indicate that IIIb/IIIc selection for all FGFR is similar in soft-tissue sarcoma of human and canine origin (Figure 3). 

Emerging information on the topic of alternative splicing control intimately links splicing of constitutive and alternative exons with transcription [76]. Evidence suggests that transcriptional modulation of promoters via various stimuli controls not only the production but also changes the exon content of the gene products [77]. Furthermore, noncoding RNAs (ncRNAs), chromatin structure, and histone modifications impact on alternative splicing regulation, suggesting that they deserve a more thorough investigation with regard to the mechanisms of splice choices [78, 79]. Epigenetic changes and ncRNAs determine not only what parts of the genome are expressed but also how they are spliced, and therefore add additional complexity on this topic. Such mechanisms may apply to FGFR splicing regulation, although reports studying the FGFR 2 and 3 promoters could not identify a correlation between a change in IIIb and IIIc splicing with transcription or epigenetic regulation [80, 81].

## 6. Novel Methods for In Vitro and In Vivo Research

Research analyzing alternative splicing events has changed over the past decade to a genome-wide scale for deciphering the regulatory networks [82–84]. Before completion of the human genome project, most approaches were based gene by gene on RT-PCR, sequencing, and comparison with existing information in sequence databases. We did use such strategies applying standard and quantitative RT-PCR to assess exon IIIb and IIIc transcripts for FGFR in human tissue material and tumor cell lines of several tumor types. For mechanistic studies using *in vitro* tumor models we have developed splice variant specific tools for both ectopic gene expression and knock down [38, 47, 49, 85].

For analyses of material from other species such as canine, we performed *in silico* analyses to locate orthologous FGFR and developed specific PCR assays that enabled the expression studies described above. In the cat, whose genome is not yet completely sequenced, we initially sequenced the four FGFRs from genomic DNA, and determined the presence of the IIIb/IIIc splice variants based upon sequence homology, direct sequencing of expressed transcripts, and splice-variant specific RT-PCR. 

Recent technical innovations have facilitated the investigation of alternative splicing at a global scale [83]. Splice-sensitive microarray platforms and deep sequencing allow quantification of large numbers of alternative splicing events. Global analyses of the targets of RBP combined with computational analyses are beginning to reveal the regulatory networks, the so-called “RNA map or RNA code” that underlies tissue-specific and developmentally regulated alternative splicing, with potential dysregulation in cancer. 

For the FGFR, a more traditional approach to study alternative splicing regulation *in vitro* and *in vivo* was based on FGFR2 minigene models and revealed cis and trans factors important for splicing regulation (reviewed in [71, 72]). Such models have been used for successful high-throughput screening of novel genes that encode splicing regulatory proteins [73]. More recently such minigene constructs have been developed to function as IIIb/IIIc alternative splicing reporters that produce different luminescent and fluorescent proteins depending on inclusion or exclusion of a specific exon [86–88]. Precise protocols for the use of such reporter in transgenic mice are already available to clarify IIIb/IIIc alternative splicing *in vivo* [89]. Similar bichromatic splicing reporters expressing two different fluorescent proteins for FGFR2 variants with potential possibilities for future modifications are briefly described (Figure 4). Such reporter systems depending on the inclusion or exclusion of the IIIc exon of FGFR2 contributed to the detection of both variants within single cancer cells *in vitro* [90, 91]. This splicing reporter system was developed and used in tumor cell models for prostate cancer in rat and was a powerful tool which helped to identify the epithelial plasticity and malignant fitness in tumor xenografts [90]. Newer splice reporter systems include the complete genomic mouse FGFR2 sequences (around 3.7 kb) of the two alternative IIIb and IIIc exons flanked by their upstream and downstream exons with introns in between and allowed the simultaneous detection of alternative exon usage *in vitro* and *in vivo* [92]. Applying this reporter system to transgenic mice and tumor cell lines revealed the evolutionally conserved switching mechanisms for the FGFR2 tissue-specific alternative splicing. Further development of this model will eventually permit assessment of the dynamics of analogous events in FGFR1 and FGFR3 processing (Figure 4). Eventually, reporter systems can be combined in tumor cell models using fluorescent proteins with multiple wavelength properties [93]. We have previously employed fluorescent reporters in the construction of chimeric proteins consisting of FGFR1 extracellular and transmembrane domains tagged to enhanced green fluorescent protein (EGFP) replacing the kinase domain. This produced a dominant negative receptor mutant while permitting identification of cells expressing the construct through the EGFP-part of the molecule [47, 49]. Based on this experience, we plan to use fluorescence reporters for analysis of IIIb/IIIc splicing in tumor cells. These tumor cell models have the advantage of monitoring the dynamics of splicing regulation with single-cell resolution, to identify essential regulation by exogeneous and endogeneous factors and to follow splicing dynamics of tumor cells during tumor progression.

## 7. Therapeutic Options

### 7.1. Targeting FGFR Splice Variants

During the past few years most FGFRs have been identified as targets for cancer therapy, whose blockade by genetic or chemical means inhibits tumor growth (see reviews [40, 94]). In addition, blockade of FGFR-dependent signaling interferes with survival pathways that cause resistance to standard therapies, and combining FGFR inhibition with standard therapeutics can result in synergistic effects [95–98]. Current inhibitor-based approaches targeting FGFRs frequently are also active against VEGFR and/or PDGFR [15, 32]. Targeting specific splice variants is not currently possible with small molecule inhibitors although it may be a promising approach in tumors that have upregulated an oncogenic FGFR-isoform. This degree of specificity can only be achieved by genetic constructs, isoform-specific antibodies, or ligand traps. Our own efforts in blocking FGFR3-isoforms in colorectal tumor cells have demonstrated that FGFR3 blockade mediated by dominant-negative mutant constructs or siRNA can be specifically targeted against FGFR3-IIIb or FGFR3-IIIc which have distinctly different biological impact depending on the choice of target [38]. 

Antibody-based therapies are being developed for FGFR3 for use in bladder cancer and multiple myeloma cell models and have been entered into clinical trials [99]. Currently available antibodies are not splice-form specific. However, splice-form specific antibodies for experimental use are commercially available, so the option for a therapeutic intervention of such high specificity exists.

**Figure 1:**
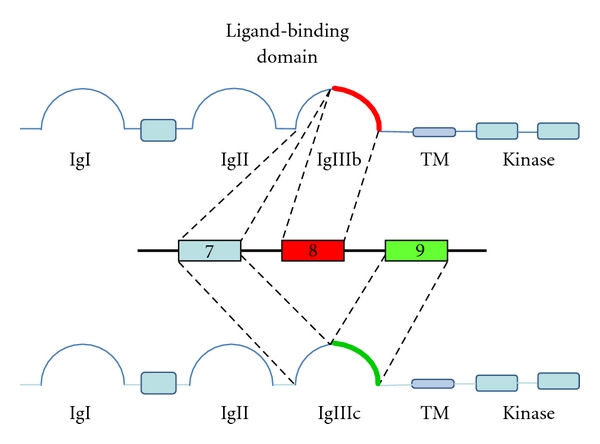
Schema splicing IIIb/IIIc. The extracellular domains of FGFRs consist of 3 Ig-like loops. The IgIII loops of FGFRs 1–3 are coded for by exons 7–9. Inclusion of exons 8 and 9 are mutually exclusive producing the IIIb and IIIc splice forms.

**Figure 2:**
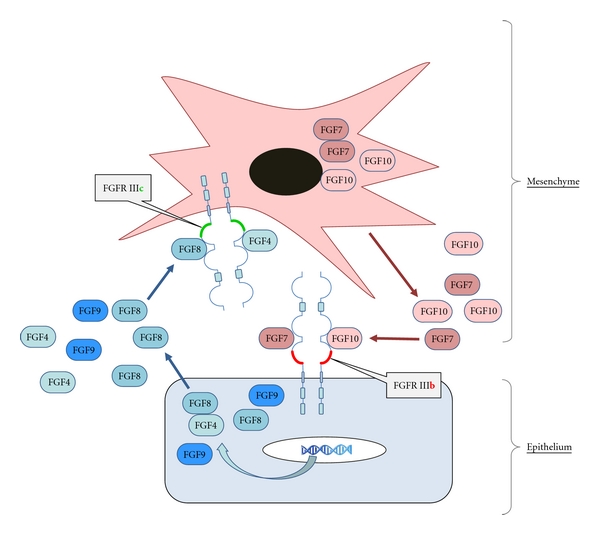
Tissue interactions mediated by IIIb/IIIc splice variants. Expression of FGFR2 variants is strictly tissue specific with the IIIb form found in the epithelium and the IIIc form in the mesenchyme. Together with expression of splice form-specific ligands in the respective complementary tissue, the splice variants mediate tissue interaction during embryonic development as well as in tissue maintenance and repair. The example FGFR2 with the IIIb-specific ligands FGF7 and 10 and the IIIc-specific ligands FGF4, 8 and 9, is involved in mouse limb development as well as in wound healing and reepithelialization in the skin [32].

**Figure 3:**
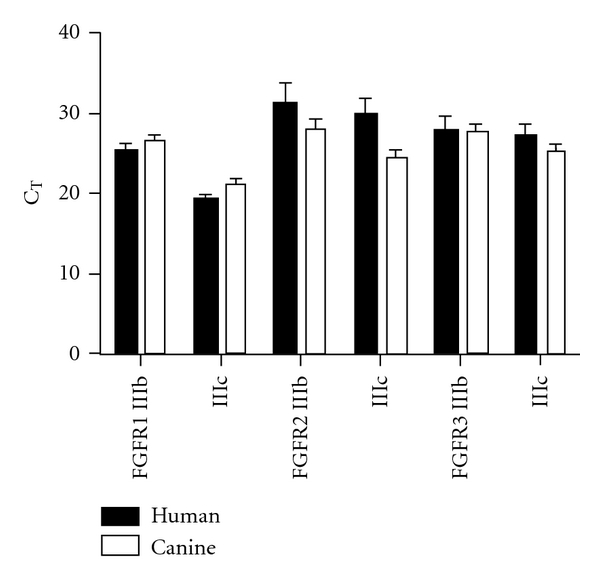
Expression patterns of FGFR1–3 IIIb and IIIc exons in human and canine STS cells. Preliminary data with quantitative real-time RT-PCR indicate that FGFR1-IIIc demonstrate lowest cycle threshold (Ct) values and thus is expressed strongest in canine STS tumors (*n* = 13) and STS cell lines (*n* = 7) originated from human STS tumors [58]. Bars and error bars represent mean with SEM.

**Figure 4:**
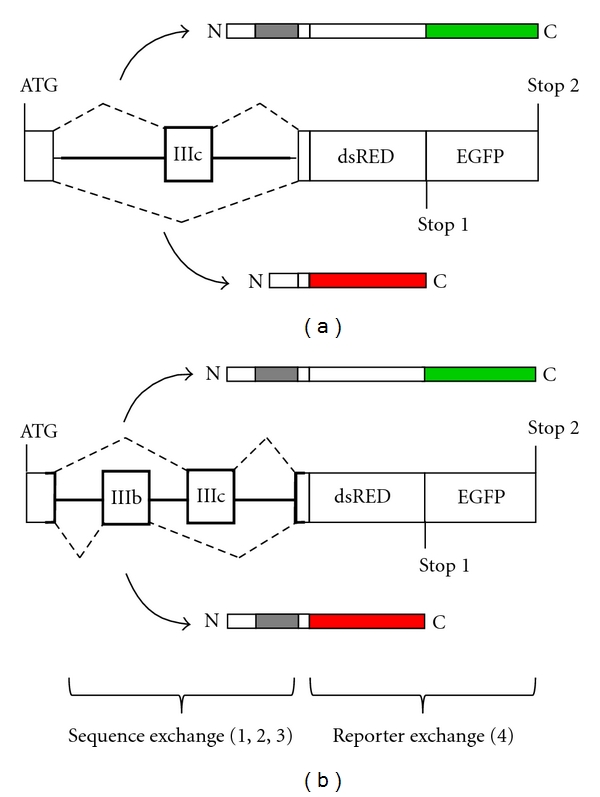
IIIb/IIIc splice reporter systems. Schematic of bichromatic fluorescence reporter constructs for IIIc (a) and IIIb/IIIc (b) analyses with adaptations from [90] and [92]. The reporter genes are positioned downstream of the splicing cassette (bold) in two different reading frames, each containing an individual stop codon. Inclusion of exon IIIc (and for b simultaneous skipping of IIIb) of FGFR2 results in a fusion protein in frame with EGFP (green) ending at stop codon 2. Skipping of this exon (and for b simultaneous inclusion of IIIb) results in DsRED (red) expression in another reading frame which contains stop codon 1. Possible adaptations of this splicing reporter system are indicated to exchange (1) species specific orthologous sequences, (2) sequences from IIIb splice variants, (3) sequences from paralogous FGFR 1 and 3, and (4) sequences for other fluorescence reporter proteins.

Ligand traps have been developed from the extracellular domains of FGFRs or from soluble splice variants. They contain the ligand-binding site of the respective receptor and trap all IIIb- or IIIc-specific FGFs that signal through it (see Figure 1). IIIc-specific ligand-traps should be suitable for interrupting those autocrine growth factors loops that are enabled by a IIIb/IIIc splice switch in carcinomas. Ligand traps have been described for FGFR4 [6] and FGFR1. The latter construct is currently in phase I clinical trials (http://clinicaltrials.gov/ identifier NCT00687505).

### 7.2. Targeting Inducers of EMT

Accumulating evidence suggests that, beyond triggering cell migration, EMT is capable of conferring stem-cell-like features such as enhanced tumor cell survival and therapy resistance. Inducing factors and pathways other than FGF include among others TGFß, EGF, IGF, and HGF (reviewed in [63]). All of these are regarded as therapeutic targets for various cancers for which targeted drugs are being developed, for example, [100]. PDGFR is another drugable tyrosine kinase that can be used to interfere with EMT [101]. 

Common down-stream mediators that have been implicated in alternative splicing regulation are the kinases in the raf-Map-kinase and the ras-PI3K-Akt pathways (reviewed in [102, 103]). The kinases have been reported to phosphorylate SR proteins in a stimulus-dependent manner affecting their subcellular location and consequently their function [104, 105]. Similar activity has also been described for protein kinase C [106] and calmodulin-dependent protein kinase [107] suggesting that multiple signaling pathways can modulate alternative splicing in a signal/cell type specific manner. 

Targeting EMT-inducing signaling pathways and cellular kinases will not be specific for splicing events due to their pleiotropic roles and interactions in cell regulation. A thorough knowledge not only of the kinases' role in splicing regulation, but also of all their additional downstream substrates will be an essential prerequisite for employing such strategies [108].

### 7.3. Targeting the Splicing Apparatus

Based on the emerging detailed information on the splicing apparatus described above, targeting on the splicing process that underlies EMT may be an innovative strategy in cancer therapy. Most importantly, it should impact not only FGFR-dependent growth and survival signals but also all the additional pro-metastatic alterations in EMT [46]. 

For this approach three different strategies seem feasible: (1) splice site modulation, (2) targeting of hnRNPs or SR proteins, and (3) targeting the spliceosome in general [109]. Splice-site modulation is the most specific approach and can be achieved directly or indirectly. Antisense oligonucleotides targeting the FGFR variant specific regulatory sequences may have direct blocking activity, similar to the intronic splicing silencer (ISS-N1) which has been shown to fully restore SMN2 exon 7 inclusion in case of spinal muscular atrophy [110]. This approach succeeded in correcting the harmful effects of a splice variant by remodeling the splice reaction and may also be useful in cancer therapy. Such a strategy depends on the identification and validation of the exact splice sites involved as they are currently available for FGFR2. However, extension of this approach to FGFRs 1 and 3, whose splice choices are regulated in a slightly different manner, may be considered as soon as the relevant sequence information is available. 

Targeting the hnRNPs or SR proteins known as splice modulators constitutes an indirect approach [109]. Several kinases are known to phosphorylate SR proteins [111]. Compounds have been identified that target these kinases and are able to modulate the splicing profile as demonstrated for Cdc2-like kinase family Clk/Sty [112, 113], for SR protein kinases SRPK1/2 [114, 115], and for topoisomerase I [116–118]. It remains to be investigated if FGFR splicing can be targeted by this strategy.

The third approach, targeting the spliceosome in general, is based on the fact that malignant cells have higher metabolic rates than normal cells and thus require increased splicing activity. Therefore, they are more sensitive to splicing modulation or inhibition [109]. A series of novel microbial compounds displaying antitumor activity [119] have recently been shown to directly target the spliceosome and modify interaction of snRNP with RNA [120, 121]. After clinical trials with the initial model compound E7107 were suspended due to problems with the structurally highly complex molecules, semisynthetic derivatives have now been developed and await further study [122]. As this strategy of directly targeting the spliceosome is not necessarily specific for tumor cells, it depends on defining drug levels that inhibit tumors but do not affect normal cells. For monitoring purposes, FGFR splicing patterns might be suitable biomarkers to assess the efficacy of these novel treatment strategies for cancer patients.

## 8. Conclusions

Alternative splicing of FGFRs producing IIIb/IIIc variants has long been known to have strong physiological and pathophysiological impact—specifically in tumor development and progression. Observations on prostate, bladder, and, lately, in colon cancer indicate that altered splice choices are related to altered cell behavior. Specifically the IIIc isoform was associated with more aggressive tumors, probably be due the broader ligand specificity of this splice forms. With FGFR2, the switch to a IIIc variant is a marker of EMT which produces invasive, metastasizing tumors.

Comparative analysis of FGFR splice patterns in a larger panel of malignancies should provide more insight in both general and tumor type specific consequences of FGFR splice choices. From this, new diagnostic/prognostic markers as well as therapeutic targets should arise.

Over the past years exciting new tools have been developed that enable high-throughput analysis of splice variants as well as a thorough investigation of the cellular mechanisms underlying FGFR splice choices. The cellular splice machinery has been characterized in sufficient detail to permit targeting not only of tumor-specific splice variants but also of the splicing process itself for cancer therapy.

Splice-reporter constructs expressing fluorescent/luminescent gene products even permit real-time observation of splice choices in single cells *in vivo* and *in vitro*. Application and further development of these new tools and technologies should help to enlighten the role of FGFR splice variants in cancer with emphasis on malignant progression.

## Figures and Tables

**Table 1:** Ligand-binding pattern of FGFR IIIb and IIIc isoforms [8].

Receptor	Ligands binding	
	IIIb	IIIc
FGFR1	FGF1, FGF2, FGF3, FGF10, FGF22	FGF1, FGF2, FGF4, FGF5, FGF6, FGF8, FGF9, FGF16, FGF17, FGF18, FGF20, FGF21, FGF23

FGFR2	FGF1, FGF3, FGF7, FGF10, FGF22	FGF1, FGF2, FGF4, FGF5, FGF6, FGF8, FGF9, FGF16, FGF17, FGF18, FGF20, FGF21, FGF23

FGFR3	FGF1, FGF9, FGF16	FGF1, FGF2, FGF4, FGF5, FGF6, FGF8, FGF9, FGF16, FGF17, FGF18, FGF20, FGF21

## Abbreviations

FGFR: Fibroblast growth factor receptors
FGF: Fibroblast growth factor
EMT: Epithelial-mesenchymal transition
MET: Mesenchymal-epithelial transition
RBP: RNA-binding proteins
SR proteins: Serine/arginine-rich proteins
hnRNP: Heterogeneous nuclear ribonucleoprotein
ESRP1/2: Epithelial splicing regulatory proteins 1 and 2
IgIII: Immunoglobulin-like loop III
EGFP: Enhance green fluorescent protein.
